# Association between Trunk Muscle Strength and Fall Risk in Older Men and Women with Lumbar Spondylosis

**DOI:** 10.3390/healthcare9050521

**Published:** 2021-04-29

**Authors:** Tadashi Ito, Yoshihito Sakai, Hideshi Sugiura, Keitaro Kawai, Yoshifumi Morita, Kazunori Yamazaki

**Affiliations:** 1 Three-Dimensional Motion Analysis Room, Aichi Prefectural Mikawa Aoitori Medical and Rehabilitation Center for Developmental Disabilities, Okazaki 444-0002, Japan; 2 Department of Physical Therapy, Graduate School of Medicine, Nagoya University, Nagoya 461-8673, Japan; hsugiura@met.nagoya-u.ac.jp; 3 Department of Orthopedic Surgery, National Center for Geriatrics and Gerontology, Obu 474-8501, Japan; jsakai@ncgg.go.jp; 4 Department of Electrical and Mechanical Engineering, Graduate School of Engineering, Nagoya Institute of Technology, Nagoya 466-8555, Japan; k.kawai.750@nitech.jp (K.K.); morita@nitech.ac.jp (Y.M.); 5 School of Health Sciences, Faculty of Clinical Engineering, Fujita Health University, Toyoake 470-1192, Japan; ymzkk@fujita-hu.ac.jp

**Keywords:** abdominal muscle, fall, muscle strength, spondylosis

## Abstract

Various factors significantly affect the risk of falls among older adults with lumbar spondylosis. However, the relationship between falls and trunk muscle strength in older men is poorly explored. Thus, we aimed to investigate the relationship between back muscle strength and fall risk in older men and women with lumbar spondylosis. Based on self-reported fall scores, 39 outpatients were classified into two groups. Back and abdominal muscle strength, among other data, were compared between the two groups. Spearman’s rank correlation analysis was used to assess the relationship between fall scores and selected variables. Back (r = −0.491, *p* = 0.002) and abdominal muscle strength (r = −0.415, *p* = 0.009) were related to the fall score. Furthermore, back and abdominal muscle strength were related to the fall score in women with a high risk of falls, whereas back muscle strength, erector spinae, and lumbar multifidus cross-sectional areas, and visual analog scale were related to the fall score in men with a high risk of falling. Back muscle strength and fall scores may be useful to assess the risk of falls in older patients with lumbar spondylosis. However, evaluating this relationship may require separate sex-specific analyses.

## 1. Introduction

Falls are a major risk for disability in the growing number of older adults in developed countries, and they are commonly observed among older adults and are related with increased disability [1]. According to previous reports, approximately 30% of older adults experience a fall annually [2,3]. Spinal disorders are associated with various symptoms, such as the difficulty of standing in an upright position, muscle weakness, decreased mobility, and increased risk of fall [4,5,6]. Lumbar spondylosis, in particular, reduces the balance function, limits day-to-day activities, and is an important cause of falls [4,5,6]. Previous studies have reported a relation between spine angle and fall among community-dwelling older adults [7,8]. A decreased L4/5 lumbar multifidus cross-sectional area and increased postural sway excursion may equally increase the risk of falling in older adults with lumbar spinal stenosis [9].

A more recent study reported that high trunk extensor muscle strength is important for preventing falls in older women [10]; meanwhile, Lohne-Seiler et al. reported an association between the trunk extensor muscle and static balance function [11]. Physical impairments, such as trunk muscle weakness, may, therefore, similarly contribute to an increased risk of falling in older adults with lumbar spondylosis [12,13,14,15]. In addition, loss of spinal lordosis (i.e., decrease in the range of spinal motion) and back muscle weakness have been reported as contributing factors to the risk of fall [12,13,14,15].

Kato et al. reported that abdominal muscle weakness was associated with falling in older women [16]; however, there are only a few reports on the relationship between falls and trunk muscle strength in older men. Moreover, few studies have focused on the association of fall risk with trunk muscle function in older men and women with lumbar spondylosis [9]. Theoretically, factors such as trunk muscle weakness or trunk muscle atrophy deterioration may also greatly affect the risk of falls in older adults with lumbar spondylosis. Therefore, this study aimed to examine the relationship between trunk muscle strength and fall risk in older men and women with lumbar spondylosis.

## 2. Materials and Methods

### 2.1. Participant Selection

A cross-sectional analysis of patients diagnosed with and conservatively treated for lumbar spondylosis was conducted at the orthopedic surgery department of a single center in Obu, Japan, between October 2018 and September 2020, which was suspended from 2 March to 25 May 2020 due to the COVID-19 emergency declaration. Three patients participated in July and August after the declaration of the state of COVID-19 emergency was lifted. The participants did not need care and were living independently. The study sample included outpatients receiving conservative treatment for spinal column stenosis, spondylitis deformans, and diabetes. For this reason, participants were undergoing basic biochemistry (HbA1c and 25(OH)D) tests (Table 1). A diagnosis of lumbar spondylosis was confirmed using the L1/2 to L4/5 area examined by magnetic resonance imaging (MRI) performed by a spine surgeon (Y.S.). The incidence of falls within the past year was recorded during an examination using a simple fall score assessment for each patient [17]. Adults aged >65 years with a high risk of falling (simple fall score >6) [17], who could walk independently, had no history of disabling arthralgia symptoms, and required no assistance in maintaining a standing posture were included. Based on the exclusion criteria, which included a simple fall score of <6 [17], history of disabling arthralgia symptoms, paralysis, spinal cord tumors, spinal infections, history of spinal or joint replacement surgery, and use of walking aids, 23 individuals were excluded. Consequently, 39 participants were enrolled in the study and comprised 19 men and 20 women with lumbar spondylosis, with a mean age of 76.5 (range, 66–85) years. Written informed consent was obtained from each participant prior to study inclusion, and all investigations were conducted in accordance with the principles stipulated in the Declaration of Helsinki. The ethics committee of our institution approved the study (IRB approval number: 586).

### 2.2. Measurement of Variables

#### Abdominal and Back Muscle Strength Assessment

The assessment was performed by an experienced medical doctor. Abdominal and back muscle strengths were determined based on the maximum isometric flexion and extension strength of the trunk muscles in the sitting position with 0° trunk extension and flexion using a digital handheld dynamometer (Isoforce GT−300, 310; OG GIKEN Co., Ltd., Okayama, Japan) [18]. Prior to muscle strength test assessment by an experienced doctor and a physical therapist, participants were allowed one trial as a learning attempt. The contraction time was within 5 s. The mean abdominal and back muscle strength values of the two trials used in the analyses were corrected by weight as follows: abdominal and back muscle strengths measured by Isoforce GT-300/weight (N/kg) [18]. 

### 2.3. Lumbar Erector Spinae and Multifidus Cross-Sectional Area

MRI was used to evaluate the cross-sectional areas of the erector spinae and lumbar multifidus muscles at the L4/5 level, and MRI scans were assessed by a radiologist [9]. Data extraction was performed using SYNAPSE (Fujifilm Medical Co., Ltd., Tokyo, Japan). The lumbar erector spinae and multifidus cross-sectional areas were calculated as cross-sectional area/weight (mm^2^/kg) [9].

### 2.4. Spine Alignment

The sagittal vertical axis (SVA) has been proposed as a measure of sagittal alignment [19] and is defined as the horizontal offset from the posterosuperior corner of S1 to the vertebral body of C7. In this study, the SVA was measured using SYNAPSE. Regional body composition was measured using dual-energy X-ray absorptiometry (DXA) (Lunar DPX, Madison, WI, USA). In addition, DXA data for the SVA were assessed by a radiologist.

### 2.5. Balance Assessments

The assessment measures were performed by an experienced medical doctor and a physiotherapist. Balance assessments were performed using a balance board (Wii; Nintendo, Kyoto, Japan) [20,21,22]. Each participant stood barefoot on the balance board, with their feet together and eyes closed, for 15 s. Participants were instructed to remain still and relaxed in the standing position with their arms hanging loosely at their sides. Postural control data represented the center of pressure (COP) signals (frequency, 100 Hz) recorded over 15 s using a microcomputer. The mean COP was defined as follows:

$$\Delta Y=Y\;\left(During\right)-\left(Pre\right)\;$$

where *Y (Pre)* and *Y (During)* were the mean values of the COP of the Y-coordinate for the first and last 15 s, respectively [9,21]. 

Further, the COP excursion of the Y-coordinate data was calculated by the root mean square values of the COP excursion displacements according to the balance assessment. 

### 2.6. Statistical Analysis

Power analysis for Spearman’s rank correlation analysis was conducted using G*Power (Heinrich Heine University Düsseldorf, Düsseldorf, Germany) to determine the optimal sample size for an alpha of 0.05, a statistical power of 0.80, a large effect size of ρ = 0.5, and two-tailed testing [23,24]. For this study, the power analysis for sample size revealed an optimal sample size of 26 participants. All statistical analyses were performed using IBM SPSS Statistics for Windows/Macintosh, ver. 24 (IBM Corp., Armonk, NY, USA). Statistical significance was accepted at *p*-value <0.05. Data were expressed as means and standard deviations or as medians (minimum and maximum values). The normal distribution of variables was confirmed using the Shapiro–Wilk test. Variable data for men and women with a high risk of falling were compared using the independent t-test (after assessing variance using Levene’s test) or Mann–Whitney U test; Spearman’s rank correlation analysis was used to assess the relationship between fall scores and the selected variables. After the analyses, the results of Spearman’s rank correlation analyses were classified according to sex. Effect sizes were considered small if r = 0.1 or −0.1, moderate if r = 0.3 or −0.3, and large if r = 0.5 or −0.5.

## 3. Results

Table 1 shows a comparison of the baseline clinical characteristics of the study participants according to sex (men, *n* = 19; women, *n* = 20). Women exhibited significantly lower abdominal muscle strength (*p* = 0.023) than men (Table 2). No significant differences were observed in the fall score, 25(OH)D, HbA1c, back muscle strength, L4/5 lumbar multifidus, and paraspinal muscle cross-sectional areas, SVA, and COP between the two groups (Table 1 and Table 2). Results of Spearman’s rank correlation analysis indicated that in older men and women with lumbar spondylosis, back muscle strength and abdominal muscle strength were significantly correlated with the fall score (Table 3). Furthermore, back muscle strength and abdominal muscle strength were related to fall score in women with a high risk of falling, whereas back muscle strength, erector spinae, and lumbar multifidus cross-sectional areas were related to fall score in men with a high risk of falling (Table 3). No correlations between other variables (SVA and COP) and fall score were noted in older men and women with lumbar spondylosis (Table 3). 

## 4. Discussion

This study found an association of high risk of falling in older women with lumbar spondylosis in abdominal and back muscle strength weakness as well as between of risk of falling in older men with lumbar spondylosis with back muscle strength weakness, a decrease of L4/5 erector spinae muscle, and lumbar multifidus. Previous studies have demonstrated that extensor and abdominal muscle weakness in older adult women result in a higher risk of falling [10,16]. In this study, Spearman’s rank correlation analysis revealed an increase in the fall score with a decrease in the abdominal and back muscle strength; however, SVA was not associated with the fall risk. This finding is consistent with the conclusion of Ito et al. [9], who stated that limited mobility due to advanced spine misalignment might not be accounted for in the fall risk assessment. These results suggest that trunk muscle weakness in older adults with lumbar spondylosis leads to falls. Meanwhile, no correlations were observed between the measured COP value and the simple fall score in this study. Furthermore, no correlations were observed between the erector spinae, lumbar multifidus muscle strength, and fall score. Back muscle strength, erector spinae, and lumbar multifidus cross-sectional areas were associated with the fall score in men, whereas abdominal and back muscle strengths were associated with the simple fall score in women. Thus, the occurrence of abdominal and back muscle weakness may be partly due to inactive muscular instability of trunk locomotion in older women with lumbar spondylosis. Meanwhile, back muscle strength weakness and decreased erector spinae and multifidus muscles in men with lumbar spondylosis could contribute to an increased risk of fall due to trunk instability. The differences between the observed correlations in men and women regarding trunk muscle function may indicate differences in trunk muscle weakness in older individuals with lumbar spondylosis. Abe et al. [25] reported that older women develop diminished mobility, whereas recent studies have reported that trunk extensor muscle or abdominal muscle strength is an important factor for fall risk in older women [10,16]. Therefore, abdominal and back muscle strengths can be used to assess the fall risk in older women with lumbar spondylosis, whereas back muscle strength, erector spinae muscle, and lumbar multifidus cross-sectional area can be used for the assessment of the fall risk in older men with lumbar spondylosis. Trunk muscle strength and cross-sectional area play important roles in body stabilization, posture maintenance, and fall risk control. Protection of the trunk muscle function is important for maintaining the ability of older men and women with lumbar spondylosis to perform day-to-day activities. Thus, these results suggest that a decline in trunk muscle strength increases the risk of falls in older men and women with lumbar spondylosis.

As the main strength of this study, only patients with a high fall risk were examined. An association was established between falling and trunk function in older women and men with lumbar spondylosis. Therefore, our results should be applicable to other patients with lumbar spondylosis.

This study had several limitations. First, it was a cross-sectional study, which did not allow prediction of the risk of falls; thus, the results might not be generalizable. Second, fall risk was categorized using a self-reported questionnaire that could be affected by social desirability bias. Third, health-related quality of life was not evaluated. Fourth, difference in the muscle and bone structures of men and women was not evaluated; assessing this could provide more information on the possible influence of fall risk. Finally, no psychological evaluation was performed in the patients. Therefore, a longitudinal study is needed in the future to clarify how improving the abdominal and back muscle strength might reduce the morbidity and mortality of falls among older patients with lumbar spondylosis.

## 5. Conclusions

This research provides insight into the relationship between trunk function and fall risk in spinal disorders. When evaluating the relationship between the risk of falling and trunk muscle strength, it may be necessary to analyze men and women separately. It is also of clinical importance to know that fall risk can differ by sex; trunk muscle weakness in older women and back muscle weakness and decreased trunk muscle cross-sectional area in older men is associated with increased fall risk. This knowledge is particularly useful for physiotherapists when implementing fall prevention and rehabilitation program for patients with lumbar spondylosis.

## Figures and Tables

**Table 1:** Characteristics of the participants.

Variables	Older Men (*n* = 19)	Older Women (*n* = 20)	*p-*Value	Effect Size (*r*)
Age (years)	76.1 [5.3]	76.2 [4.4]	0.926	0.02
Height (cm)	162.2 [5.8]	149.8 [5.4]	0.0001	0.8
Weight (kg)	66.7 [11.7]	58.2 [11.8]	0.003	0.4
BMI (kg/m^2^)	25.2 [3.4]	25.9 [4.8]	0.626	0.1
Fall score (points)	6 (6–13)	7 (6–13)	0.728	−0.1
25(OH)D	16.9 [3.6]	14.1 [5.6]	0.075	0.3
HbA1c	5.8(5.4–7.3)	5.9(5.5–6.9)	0.832	−0.03

Data are presented as the mean (standard deviation) or median value (minimum and maximum values). *p-*values for height and weight were calculated using the Mann–Whitney U test; the remaining *p*-values were generated using an independent t-test. BMI, body mass index.

**Table 2:** Functional outcomes of the participants.

Variables	Older Men (*n* = 19)	Older Women (*n* = 20)	*p-*Value	Effect Size (*r*)
Abdominal muscle strength (N/kg)	1.7 [0.4]	1.5 [0.4]	0.023	0.4
Back muscle strength (N/kg)	2.4 [0.8]	2.0 [0.5]	0.071	0.3
L4/5 erector spinae cross-sectional area (mm^2^/kg)	27.5 [7.2]	28.1 [7.4]	0.807	0.04
L4/5 lumbar multifidus cross-sectional area (mm^2^/kg)	12.8 [3.6]	10.4 [4.0]	0.061	0.3
SVA (mm)	79.1 [42.6]	80.5 [35.5]	0.915	0.02
Center of pressure (mm)	7.9 (4.8–18.1)	6.7 (2.0–14.9)	0.054	0.3

Data are presented as the mean (standard deviation) or median value (minimum and maximum values). All *p*-values were generated using the independent t-test. SVA, sagittal vertical axis.

**Table 3:** Correlation between the fall score and abdominal and back muscle strength and L4/5 erector spinae and lumbar multifidus cross-sectional areas.

Variables	All Participants	Men	Women	*p*-Value
Abdominal muscle strength	−0.415	−0.297	−0.507	A: 0.009 *
				M: 0.217
				W: 0.022 *
Back muscle strength	−0.491	−0.457	−0.541	A: 0.002 *
				M: 0.049 *
				W: 0.014 *
L4/5 erector spinae cross-sectional area	−0.246	−0.534	0.041	A: 0.131
				M: 0.019 *
				W: 0.862
L4/5 lumbar multifidus cross-sectional area	−0.225	−0.548	0.213	A: 0.169
				M: 0.015 *
				W: 0.0366
SVA	0.093	−0.076	0.244	A: 0.572
				M: 0.759
				W: 0.299
Center of pressure	−0.005	0.135	−0.084	A: 0.974
				M: 0.581
				W: 0.723

A, all participants; M, men; W, women; SVA, sagittal vertical axis. Back muscle strength and abdominal muscle strength were related to the fall score in women with a high risk of falling, whereas back muscle strength, erector spinae, and lumbar multifidus cross-sectional area were related to the fall score in men with a high risk of falling. * values in superscript are variables with a significant correlation (*p* < 0.05).

## Data Availability

All of the relevant data are presented within the manuscript. All data are available from the authors on request.
